# Comparison of clinicians’ and researchers’ ratings of proposed diagnostic criteria for compulsive buying-shopping disorder within a Delphi study

**DOI:** 10.1371/journal.pone.0283978

**Published:** 2023-04-04

**Authors:** Nora M. Laskowski, Patrick Trotzke, Kathina Ali, Dan B. Fassnacht, Mike Kyrios, Michael Häder, Astrid Müller

**Affiliations:** 1 Medical Faculty, University Clinic for Psychosomatic Medicine and Psychotherapy, Campus East-Westphalia, Ruhr-University Bochum, Luebbecke, Germany; 2 Department of Psychosomatic Medicine and Psychotherapy, Hannover Medical School, Hannover, Germany; 3 General Psychology, Cognition and Center for Behavioral Addiction Research (CeBAR), University of Duisburg-Essen, Duisburg, Germany; 4 IU International University of Applied Sciences, Erfurt, Germany; 5 College of Education, Psychology and Social Work, Flinders University, Adelaide, Australia; 6 Research School of Psychology, The Australian National University, Canberra, Australia; 7 Institute of Sociology, Technical University of Dresden, Dresden, Germany; Yale University, UNITED STATES

## Abstract

Diagnostic criteria for compulsive buying shopping disorder were recently proposed based on a Delphi consensus study including 138 experts from 35 countries. The present study represents a secondary analysis of those data. To provide further support for the validity of expert responses in the Delphi study, the sample was retrospectively divided into *clinician* and *researcher* subgroups. The two groups were compared with respect to demographic variables, their importance ratings of clinical features, possible diagnostic criteria, differential diagnoses and specifiers of compulsive buying shopping disorder. *Researchers* reported less years of treating/assessing individuals with compulsive buying shopping disorder and stated that they have treated/assessed individuals with compulsive buying shopping disorder less often in the last 12 months than *clinicians*. Responses from the two groups concerning the importance ratings of possible diagnostic criteria of compulsive buying shopping disorder converged with only few minor differences with small to moderate group effects. However, even for those criteria, the consensus threshold (≥75% agreement with the proposed criterion) was reached in both groups. The lack of differences in the responses of the two groups indicates good validity for the proposed diagnostic criteria. Future research should address the clinical applicability and diagnostic validity of the criteria.

## Introduction

The consumption of consumer goods can become pathological if it is associated with preoccupations and cravings towards buying/shopping, characterized by diminished control over searching for products and purchasing, has gained priority in a person’s life, and is continued or even escalated despite negative consequences [1–4]. The latter include significant clinical distress, financial, family, occupational and social problems, reduced quality of life and impairments in important areas of functioning [5,6]. With a pooled estimated prevalence of about 5% in the general population across different cultures [7], the importance of excessive, maladaptive buying and shopping activities as a public health challenge becomes more and more apparent.

Pathological buying has been mentioned in the literature more than 100 years ago as “oniomania” [8]. However, its recognition as a separate mental disorder is still pending. Due to the failures of impulse control and other similarities with impulse control disorders (e.g., increasing tension prior to a shopping episode, which is relieved while shopping/buying), the classification as an impulse control disorder has been preferred for a long time [9], resulting in the term “compulsive buying-shopping disorder” as an example of “other specific impulse control disorder” in the coding tool of the 11^th^ revision of the International Classification of Diseases (ICD-11; [4]). In accordance with the ICD-11 [4], we adopt the term “compulsive buying-shopping disorder” (CBSD) thereinafter for the purposes of this paper. It is, however, important to note that alternatively to the ICD-11 approach, CBSD may be considered an “other specific disorder due to addictive behaviors” [4] due to shared neurocognitive characteristics with behavioral addictions (i.e., gambling disorder, gaming disorder; [10]). These include reward sensitivity, cue-induced craving, reduced inhibitory control and decision-making deficits [10–14].

More than 25 years ago, McElroy et al. proposed preliminary operational criteria for CBSD, derived from the DSM-III-R criteria for obsessive compulsive, substance use and gambling disorders [2]. However, their clinical utility, reliability and validity were never specified or evaluated systematically. Recently, diagnostic criteria for CBSD were proposed based on a Delphi consensus study [1,3]. This study was a collaborative project between Australian (Australian National University, Canberra; Flinders University, Adelaide) and German scientists (Hannover Medical School, University of Duisburg-Essen, Technical University of Dresden). The Delphi methodology was originally developed as a prognostic or forecasting tool, but has since been used in many other areas, including mental health [15] with a focus primarily on the process of reaching consensus. The Delphi study mentioned above included a two-stage online survey with international experts in the field of CBSD. Based on a literature search, first and last authors of scientific publications on CBSD were identified and invited to participate in the survey. The items of the questionnaire were based on literature searches, clinical and scientific expertise of the study team and DSM-5 criteria [16] of phenomenologically similar disorders (e.g., gambling, gaming and hoarding disorder). The questionnaire used in the first wave of the survey (12/2018-03/2019) contained 37 items on possible diagnostic criteria of CBSD. In addition, there were questions on terminology, classification, specifications, differential diagnoses, and frequency of buying episodes. Furthermore, some control items were included to test for divergent validity (e.g., questions on symptoms that are typical for other disorders but not for CBSD). The experts were asked to rate each item on a 4-point scale in terms of its importance for the diagnosis of CBSD (“irrelevant”, “peripheral”, “important”, “essential” or “I don’t know”). They were also asked how sure they were about their answer (“not at all sure”, “uncertain”, “neither certain nor uncertain”, “certain”, “absolutely sure”, “I don’t know”). To give experts the opportunity to provide additional information, open-ended questions were placed in several sections. The survey concluded with questions on experts’ sociodemographic characteristics, English language proficiency, and (clinical) experience with CBSD. Valid responses were available from 138 experts who came from 35 countries. After evaluation of the first wave of the survey, a questionnaire with a total of 79 items was developed for the second wave (08/2019–11/2019) and sent to all experts who had participated in the first wave. The questionnaire contained all items for which no consensus was reached in the first wave. Each of these items was accompanied by anonymized feedback about the group ratings of the first wave. In addition, some new items were added based on suggestions (to open-ended questions) of the participants. In the second wave of the survey, 102 experts from 26 countries participated. In line with other Delphi studies, a consensus criterion of 75% agreement or disagreement with the criteria proposed was used in the 1^st^ and 2^nd^ wave [17]. A more detailed description of the procedure, results and their interpretation can be found in Müller, Laskowski, et al. [3]. The findings in that publication refer to the entire expert panel as well as to a subgroup of so called “master experts”. This subsample had indicated greater self-reported expertise and knowledge with respect to CBSD. All analyses were performed for the entire sample and for the subgroup of master experts to double-check the validity of the results [3,18].

The present investigation represents a secondary analysis of data collected within the Delphi study [3] by applying an alternative way to support previous findings. Instead of creating a group of self-identified “master experts”, the expert sample was divided into *clinicians* and *researchers*. This helped determine whether *clinicians* or *researchers* were underrepresented in the Delphi study. This approach further provided the opportunity to address the question whether *clinicians* and *researchers* differed in their judgment of potential diagnostic criteria and other characteristics of CBSD. The analysis was based on presumed differences between the two groups. Thus, it may have been possible that clinical experience was higher among *clinicians* because they had actually worked with patients experiencing CBSD. Instead, *researchers* may have assessed items in a more theoretical or model-oriented manner than *clinicians*. Consequently, the professional background of participants might have affected their responses.

Due to the explorative nature of the analyses, no formal hypotheses regarding group differences were drawn, with the exception of the expected higher self-reported experience in assessing and treating patients experiencing CBSD among *clinicians* compared to *researchers*.

## Materials and methods

### Grouping *researchers* and *clinicians*

The flow chart of the Delphi study expert selection can be found in the previously published Delphi study results [3]. All experts who participated in the Delphi study were considered for the formation of the groups (*N* = 130). The grouping of experts into the *researcher* (*n* = 56) and *clinician* (*n* = 74) subsamples was made based on the following open-ended question that was administered in the first round of the Delphi study: “What is your concrete field of work?”. No response options were provided for this question. The first and last authors (NML, AM) then assigned the experts’ answers to one of the two groups (*researchers* or *clinicians*; consensus was met κ = 1). All answers to these questions could be clearly assigned to one of the two groups (e.g., “postdoctoral researcher working in behavioral addictions” for the *researcher* group and “drug and behavioral addiction treatment” for the *clinician* group).

### Statistical analysis

This is a secondary data analysis, ethical approval for data collection was obtained from the review board of Hannover Medical School, Germany (No. 8081-BO-K-2018). Before commencing the survey, participants provided digital informed consent.

First, the group of *clinicians* and *researchers* were compared with respect to sociodemographic variables and their self-reported experience with respect to the assessment and treatment of patients with CBSD. Next, their responses (approval vs. rejection; answers to open questions) regarding general aspects (e.g., terminology, classification), phenomenological characteristics (i.e., diagnostic criteria) and differential diagnoses of CBSD were examined. The initial Delphi study also included suggestions for potential specifiers, e.g., “‘buying (i.e., purchasing items)’ vs. ‘shopping (i.e., window shopping, browsing)’ should be considered as a specifier for buying-shopping disorder”. However, specifiers missed consensus amongst the “master experts” [3]. In view of the ICD-11 approach for gaming and gambling disorders [4], the negative consequences of addictive online behaviors [19] and the generalization of in-person CBSD to the e-marketplace which has been exacerbated during the COVID pandemic [20–22], experts’ ratings of the specifier “predominantly offline” and “predominantly online” were examined again in more detail. An additional focus was placed on all criteria that referred to excessive hoarding in association with CBSD, regardless of whether the expert panel had reached consensus to include or exclude those characteristics within the Delphi study. These items caused great uncertainty among experts within the Delphi study [3]. The exploration of possible differences between *clinicians’* and *researchers’* responses could provide further insights into consensus on those items. Statistical analyses were conducted using SPSS version 26 [23]. C*linicians* were compared to *researchers* with respect to demographic variables and importance ratings (i.e., frequencies of agreement or disagreement) using χ^2^- or Fisher-Exact-tests (when expected cell frequencies < 5). For all tests, Cramer’s V *(V)* was calculated as an effect size for variables with >2 categories, φ coefficients are reported as effect sizes for variables with ≤2 categories. Values of *V*/φ<0.1 are considered as small effects, *V*/φ<0.3 as moderate effects, and *V*/φ> 0.5 as large effects [24]. Age differences were analyzed using the Mann-Whitney-U-Test, and the partial ɳ^2^ is reported as effect size. In addition, Cohen’s kappa (κ) was used as the degree of agreement between the two groups [25]. Values below 0.2 were considered slight, values between 0.21 and 0.4 fair, values between 0.41 and 0.60 moderate, and values from 0.61 to 0.80 substantial. Values above 0.81 were considered almost perfect [26].

Post hoc, we specified differences between groups (*clinicians* vs. *researchers*) by controlling for demographic variables with hierarchical binary logistic regressions. Effect sizes for the models were indicated by Nagelkerkes *R*^2^ and for the coefficients by Wald statistic (*W*). The significance level for α was set at p < .01 in order to correct for multiple comparisons.

## Results

### Sample characteristics and group comparison of sociodemographic variables

The group *researcher* contained 56 experts and the group *clinician* 74 experts. Sample characteristics and group comparisons are presented in Table 1. The missing values are given for overview purposes only and have not been included in the calculations. Regarding the area of profession, *clinicians* included more experts from the field of medicine than *researchers*, while *researchers* consisted of more experts from the field of consumer research than *clinicians*. Another group difference can be seen in the work experience: The group of *researchers* reported less years of treating/assessing individuals with CBSD than *clinicians* which makes sense due to the nature of these professions. In line with that, *researchers* stated that they have treated/assessed individuals with CBSD less often in the last 12 months than *clinicians*. There were no group differences regarding the country of work, level of proficiency of English, age or gender.

**Table 1:** Description of the expert panel.

Question	*Group*		Statistics
	Researcher (*N* = 56) *n* (%)	Clinician (*N* = 74) *n* (%)	
“Which area ‘best’ describes your profession?” Psychology Medicine Public Health Consumer Research Other	30 (53.6) 4 (7.1) 1 (1.8) 18 (32.1) 3 (5.4)	33 (44.6) 37 (50.0) 1 (1.4) 1 (1.4) 2 (2.7)	*p* < .001^a^*; *V* = 0.58
“In which country do you work?” USA Germany Spain UK Italy Canada Denmark The Netherlands Australia Brazil France Turkey Switzerland Other *Missing Data*	13 (23.6) 8 (14.5) 4 (7.3) 4 (7.3) 3 (5.5) 2 (3.6) 2 (3.6) 2 (3.6) 1 (1.8) - 1 (1.8) 1 (1.8) 1 (1.8) 13 (23.6)^b^ *1*	10 (13.5) 11 (14.9) 6 (8.1) 2 (2.7) 7 (9.5) 3 (4.1) - 4 (5.4) 6 (8.1) 6 (8.1) 6 (8.1) 3 (4.1) 2 (2.7) 8 (10.8)^c^ ^-^	*p* = .088^a^; *V* = 0.56
“How would you describe your level of knowledge and experience related to BSD?” A little A moderate amount Quite a bit A great deal	5 (8.9) 23 (41.1) 20 (35.7) 8 (14.3)	5 (6.8) 14 (18.9) 32 (43.2) 23 (32.1)	χ^2^(3) = 9.91, *p* = .019; *V* = 0.28
“For how many years have you been treating or assessing individuals with BSD?” 0 years ≤ 5 years 6–10 years 11–15 years 16–20 years ≥ 21 years *Missing Data*	37 (67.3) 9 (16.4) 4 (7.3) 1 (1.8) 1 (1.8) 3 (5.5) *1*	10 (13.9) 16 (22.2) 20 (27.8) 5 (6.9) 16 (22.2) 5 (6.9) *2*	*p* < .001^a^*; *V* = 0.58
“In the past 12 months, how often have you treated or assessed individuals with BSD?” Never Rarely: ≤ once per month Sometimes: a couple of times a month Often: about once a week Very frequently: multiple times per week *Missing Data*	42 (79.2) 4 (7.5) 5 (9.4) 2 (3.8) - *3*	17 (23.0) 25 (33.8) 18 (24.3) 11 (14.9) 1 (4.1) -	*p* < .001^a^*; *V* = 0.56
“Please indicate your level of proficiency in English.” Elementary proficiency Limited working proficiency Professional working proficiency Full professional proficiency Native or bilingual proficiency	1 (1.8) 2 (3.6) 17 (30.4) 14 (25.0) 22 (39.3)	1 (1.4) 7 (9.5) 25 (33.8) 23 (31.1) 18 (24.3)	*p* = .313^a^; *V* = 0.19
Age (“What is your year of birth?”) *Mean* (*SD*) *Median* (*Range*)	47.37 (11.25) 47 (28–72)	50.69 (10.96) 50 (29–79)	*U* = 1497.0, *z* = -1.74, *p* = .082; η^2^ = 0,02
“Gender” Male Female *Missing Data*	30 (54.5) 25 (45.5) *1*	41 (55.4) 33 (44.6)	χ^2^(1) = 0.01, *p*>.999; *φ* = 0.01

Note. BSD = buying-shopping disorder

^a^Fisher-Exact-Test; ^b^Other (*n* = 1): China, Hungary, Iceland, Norway, Portugal, Singapore, South Korea, Taiwan, UAE, Several Countries (*n* = 4); ^c^Other (*n* = 1): Austria, Belgium, Ecuador, Iran, Israel, Luxembourg, Poland, Russia

*p < .01.

### Group comparison of responses regarding general features of CBSD

Table 2 contains the group comparison of ratings regarding classification, terminology, and frequency of buying-shopping episodes. No group differences in the answers to those questions were found, whereas Cohen’s kappa indicates moderate values of agreement.

**Table 2:** Group comparison regarding general questions.

Question	*Group*		Statistic
	Researcher (*N* = 56) *n* (%)	Clinician (*N* = 74) *n* (%)	
“If you believe that BSD is a distinct psychiatric diagnosis, how should it be classified?” Impulse Control Disorder Disorder due to Addictive Behavior Obsessive Compulsive Disorder *Missing Data*	2 (21.4) 21 (75.0) 1 (3.6) *28*	10 (21.7) 35 (76.1) 1 (2.2) *28*	*p*>.999^a^; *V* = 0.04, κ = 0.56
“If you believe that BSD is a distinct psychiatric diagnosis, what should it be called?” Compulsive Buying Disorder Buying-Shopping Disorder Shopping Disorder *Missing Data*	17 (60.7) 11 (39.3) - *28*	25 (51.0) 20 (40.8) 4 (8.2) *25*	*p* = .385^a^; *V* = 0.18, κ = 0.56
“Do you think a frequency (of buying-shopping episodes) item is needed for the diagnosis of BSD?" No Yes *Missing Data*	22 (64.7) 12 (35.3) *22*	38 (69.1) 17 (30.9) *19*	χ^2^(1) = 0.18, *p* = .668; *φ* = -0.05, κ = 0.57

Note. BSD = buying-shopping disorder

^a^Fisher-Exact-Test.

### Group comparison of responses regarding consented phenomenological characteristics and differential diagnoses of CBSD

Another aim of the study was to examine whether *clinicians* and *researchers* differed in how they rated the consented diagnostic criteria for CBSD in the Delphi study. As shown in Table 3, group differences emerged only for the criterion: “Repeated unsuccessful efforts to reduce or control buying/shopping” and differential diagnosis “Excessive buying/shopping does not occur exclusively during a period of mania/hypomania.”, with *clinicians* more often agreeing with these characteristics than *researchers*. However, the items were consented by both groups (i.e., in both groups more than 75% considered these items to be important) and the magnitude of the observed group effects was only small to moderate. Cohen’s kappa indicates a moderate to substantial degree of concordance between the two groups.

**Table 3:** Group comparison regarding consented phenomenological characteristics and differential diagnoses of CBSD.

Phenomenological characteristic	Group		Statistics
	Researcher (*N* = 56) *n* (%)	Clinician (*N* = 74) *n* (%)	
“Can a diagnosis of BSD co-occur in the context of ‘borderline and/or other personality disorders’?” No Yes	5 (18.5) 22 (81.5)	5 (9.1) 50 (90.9)	*p* = .285^a^; *φ* = 0.14, κ = 0.56
“How important do you think […] is/are in BSD?”			
Preoccupations Irrelevant/Peripheral Important/Essential	8 (14.8) 46 (85.2)	11 (15.1) 62 (84.9)	χ^2^(1) = 0.002, *p*>.999; *φ* = -0.004, κ = 0.67
Obsessions Irrelevant/Peripheral Important/Essential	6 (16.7) 30 (83.3)	10 (17.2) 48 (82.8)	χ^2^(1) = 0.005, *p*>.999; *φ* = -0.007, κ = 0.59
Inability to stop thinking Irrelevant/Peripheral Important/Essential	7 (12.5) 49 (87.5)	5 (6.8) 69 (93.2)	χ^2^(1) = 1.26, *p* = .263; *φ* = 0.098, κ = 0.68
Repetitive intrusive thoughts Irrelevant/Peripheral Important/Essential	5 (13.9) 31 (86.1)	10 (16.9) 49 (83.1)	χ^2^(1) = 0.16, *p* = .691; *φ* = -0.04, κ = 0.59
Repetitive impulses to buy/shop Irrelevant/Peripheral Important/Essential	3 (5.4) 53 (94.6)	0 74 (100)	*p* = .077^a^; *φ* = 0.18, κ = 0.68
Maintenance or escalation of buying/shopping despite negative consequences Irrelevant/Peripheral Important/Essential	3 (8.3) 33 (91.7)	1 (1.7) 58 (89.3)	*p* = .151^a^; *φ* = 0.16, κ = 0.59
Returning purchased items without utilizing them Irrelevant/Peripheral Important/Essential	31 (88.6) 4 (11.4)	44 (77.2) 13 (22.8)	χ^2^(1) = 1.86, *p* = .172; *φ* = 0.14, κ = 0.58
“How important is […] in BSD?”			
Strong or irresistible desire to buy/shop Irrelevant/Peripheral Important/Essential	2 (3.6) 54 (96.4)	0 74 (100)	*p* = .184^a^; *φ* = 0.14, κ = 0.68
Irresistible urge to engage in buying/shopping activities Irrelevant/Peripheral Important/Essential	1 (1.8) 55 (98.2)	1 (.4) 73 (98.6)	*p*>.999^a^; *φ* = 0.02, κ = 0.69
Craving for the high while buying/shopping Irrelevant/Peripheral Important/Essential	10 (17.9) 46 (82.1)	12 (16.4) 61 (83.6)	χ^2^(1) = 0.05, *p>*.999; *φ* = 0.02, κ = 0.68
Buying/Shopping is used to generate positive emotions Irrelevant/Peripheral Important/Essential	9 (17.0) 44 (83.0)	10 (13.5) 64 (86.5)	χ^2^(1) = 0.30, *p* = .589; *φ* = 0.05, κ = 0.66
Buying/shopping is used to regulate negative emotions Irrelevant/Peripheral Important/Essential	8 (14.5) 47 (85.5)	12 (16.7) 60 (83.3)	χ^2^(1) = 0.11, *p* = .745; *φ* = -0.03, κ = 0.67
Craving for relief from negative internal states while buying/shopping Irrelevant/Peripheral Important/Essential	1 (2.9) 34 (97.1)	3 (5.1) 56 (94.9)	*p*>.999^a^; *φ* = -0.05, κ = 0.58
Loss of control/self-control Irrelevant/Peripheral Important/Essential	3 (5.4) 53 (94.6)	1 (1.4) 73 (98.6)	*p* = .314^a^; *φ* = 0.12, κ = 0.68
Spending much more time buying/shopping than intended Irrelevant/Peripheral Important/Essential	14 (25.2) 41 (74.5)	12 (16.2) 62 (83.8)	χ^2^(1) = 1.67, *p* = .196; *φ* = 0.11, κ = 0.67
Buying many more things or spending more than necessarily needed/intended Irrelevant/Peripheral Important/Essential	9 (16.1) 47 (83.9)	6 (8.1) 86 (91.9)	χ^2^(1) = 1.98, *p* = .159; *φ* = 0.11, κ = 0.68
Buying many more things than can be afforded Irrelevant/Peripheral Important/Essential	5 (13.9) 31 (86.1)	10 (17.5) 47 (82.5)	χ^2^(1) = 0.22, *p* = .641; *φ* = -0.05, κ = 0.59
Buying/shopping something ‘on the spur of the moment’ Irrelevant/Peripheral Important/Essential	3 (8.6) 32 (91.4)	8 (13.6) 51 (86.4)	*p* = .529^a^; *φ* = -0.08, κ = 0.58
Repeated unsuccessful efforts to stop thinking about buying/shopping Irrelevant/Peripheral Important/Essential	5 (13.9) 31 (86.1)	10 (16.9) 49 (83.1)	χ^2^(1) = 0.16, *p* = .691; *φ* = -0.04, κ = 0.59
Repeated unsuccessful efforts to reduce or control buying/shopping Irrelevant/Peripheral Important/Essential	12 (22.2) 42 (77.8)	4 (5.5) 69 (94.5)	χ^2^(1) = 7.90, *p* = .005*; *φ* = 0.25, κ = 0.67
“Reduction or cessation of buying/shopping results in […].”			
Emotional symptoms Irrelevant/Peripheral Important/Essential	11 (19.6) 45 (80.4)	14 (19.2) 59 (80.8)	χ^2^(1) = 0.004, *p* = .947; *φ* = 0.01, κ = 0.68
Cognitive symptoms Irrelevant/Peripheral Important/Essential	3 (8.3) 33 (91.7)	8 (13.8) 50 (86.2)	*p* = .522^a^; *φ* = -0.08, κ = 0.59
“Buying/shopping preoccupations, obsessions, impulses or behaviors result in […].”			
Clinically significant distress Irrelevant/Peripheral Important/Essential	8 (14.8) 46 (85.2)	4 (5.4) 70 (94.6)	χ^2^(1) = 3.25, *p* = .071; *φ* = 0.16, κ = 0.70
Negative feelings Irrelevant/Peripheral Important/Essential	7 (12.5) 49 (87.5)	10 (13.5) 64 (86.5)	χ^2^(1) = 0.03, *p* = .865; *φ* = -0.02, κ = 0.68
Impairment in social life Irrelevant/Peripheral Important/Essential	6 (10.9) 49 (89.1)	6 (8.2) 67 (91.8)	χ^2^(1) = 0.27, *p* = .605; *φ* = 0.05, κ = 0.67
Impairment in occupational areas Irrelevant/Peripheral Important/Essential	3 (8.6) 32 (91.4)	10 (17.5) 47 (82.5)	*p* = .357^a^; *φ* = -0.13, κ = 0.62
Impairment in other important areas of functioning Irrelevant/Peripheral Important/Essential	9 (17.0) 44 (83.0)	11 (15.1) 62 (84.9)	χ^2^(1) = 0.08, *p* = .772; *φ* = 0.03, κ = 0.66
Financial difficulties including indebtedness, bankruptcy Irrelevant/Peripheral Important/Essential	13 (23.6) 42 (76.4)	13 (17.8) 60 (82.2	χ^2^(1) = 0.66, *p* = .417; *φ* = 0.07, κ = 0.67
Loss of interest in other life activities and hobbies Irrelevant/Peripheral Important/Essentia	5 (13.9) 31 (86.1)	5 (8.6) 53 (91.4)	*p* = .499^a^*; φ* = 0.08, κ = 0.59
Lying to others about buying/shopping Irrelevant/Peripheral Important/Essential	10 (18.5) 44 (81.5)	19 (26.0) 54 (74.0)	χ^2^(1) = 0.99, *p* = .319; *φ* = -0.09, κ = 0.67
Delinquency Irrelevant/Peripheral Important/Essential	28 (80.0) 7 (20.0)	45 (76.3) 14 (23.7)	χ^2^(1) = 0.18, *p* = .675; *φ* = 0.04, κ = 0.58
Differential diagnoses: “Excessive buying/shopping does not occur exclusively during a period of […].”			
Mania/hypomania Irrelevant/Peripheral Important/Essential	11 (23.4) 36 (76.6)	1 (1.4) 73 (98.6)	*p* < .001^a^*; *φ* = 0.36, κ = 0.62
Psychosis Irrelevant/Peripheral Important/Essential	1 (2.9) 33 (97.1)	4 (6.9) 54 (93.1)	*p* = .648^a^; *φ* = 0.08, κ = 0.57
Organic psychosyndrome (OPS) Irrelevant/Peripheral Important/Essential	10 (23.8) 32 (76.2)	9 (13.0) 60 (87.0)	χ^2^(1) = 2.13, *p* = .144; *φ* = 0.14, κ = 0.69

Note. BSD = buying-shopping disorder

^a^Fisher-Exact-Test

*p < .01.

### Group comparison of responses regarding the suggested specifier ‘predominantly offline’ vs. ‘predominantly online’

There were no significant differences between the groups. Both expert groups did not consent with the suggested specifier “‘predominantly offline’ vs. ‘predominantly online’” (i.e., neither group gave ≥75% agreement). Among *researchers*, 18 (52.9%) stated it was irrelevant/peripheral, compared to 27 (46.6%) *clinicians*. Conversely, 31 *clinicians* (53.4%) and 16 (47.1%) *researchers* indicated that this specifier should be considered. There was no group difference and moderate conformity (χ^2^(1) = 0.35, *p* = .554; *φ* = 0.062, κ = 0.57).

### Group comparison of responses regarding criteria referring to excessive hoarding

Experts’ responses to suggested criteria referring to excessive hoarding of purchased consumer goods were considered separately, irrespective of the presence or absence of consensus in the Delphi study. The experts admitted substantial uncertainties with respect to their ratings of hoarding-related criteria within the initial Delphi study [3]. In this secondary analysis, no significant differences between *clinicians’* and *researchers’* importance ratings were found (Table 4). Both groups rated the criterion “excessive buying/shopping does not occur exclusively during ‘acquisition of purchased or free items as a result of hoarding disorder’” as important or essential for the diagnosis of CBSD (i.e., the consensus criterion of ≥75% agreement was reached; Table 4). Of note, however, consensus for the specifier “‘with vs. without hoarding disorder’” was reached by *clinicians* but not by *researchers*. All Cohen’s kappa values indicate a moderate to substantial degree of concordance.

**Table 4:** Group comparison regarding hoarding items.

Phenomenological characteristic	Group		Statistic
	Researcher (*N* = 56) *n* (%)	Clinicians (*N* = 74) *n* (%)	
“Excessive buying/shopping does not occur exclusively during ‘acquisition of purchased or free items as a result of hoarding disorder’.” Irrelevant/Peripheral Important/Essential *Missing Data*	8 (24.2) 25 (75.8*) *23*	13 (24.5) 40 (75.5*) *21*	χ^2^(1) <0.01, *p* = .976; *φ*<-0.01, κ = 0.57
“Can a diagnosis of BSD co-occur in the context of ‘hoarding disorder’?” No Yes *Missing Data*	6 (22.2) 21 (77.8*) *29*	12 (25.5) 35 (74.5) *27*	χ^2^(1) = 0.10, *p* = .749; *φ* = -0.04, κ = 0.55
Specifier			
“‘With vs. without hoarding disorder’ should be considered as a specifier for BSD.” Irrelevant/Peripheral Important/Essential *Missing Data*	10 (29.4) 24 (70.6) *22*	12 (21.8) 43 (78.2*) *19*	χ^2^(1) = 0.65, *p* = .420; *φ* = 0.09, κ = 0.66
“‘With difficulty discarding vs. without difficulty discarding’ should be considered as a specifier for BSD.” Irrelevant/Peripheral Important/Essential *Missing Data*	22 (68.8) 10 (31.3) *24*	29 (54.7) 24 (45.3) *21*	χ^2^(1) = 1.64, *p* = .201; *φ* = 0.14, κ = 0.63

Note. BSD = buying-shopping disorder

*Consensus is reached on agreement/disagreement above 75% for both groups.

### Post hoc verification for the significant group differences controlled by demographic variables

To analyze the differences between *clinicians* and *researchers*, we controlled for significant demographic variables with hierarchical binary logistic regressions. The analyses were conducted for the two items where the groups differed (criterion “repeated unsuccessful efforts to reduce or control buying/shopping” and differential diagnosis “excessive buying/shopping does not occur exclusively during a period of mania/hypomania”; see Table 3). Based on the assumption that the main area of profession and assessment/treatment experience could have influenced experts’ responses, we adjusted the analyses for those variables. To account for assessment/treatment experience, experts’ answers to the question “In the past 12 months, how often have you treated or assessed individuals with BSD?” were used (see Table 1). We only chose these two control variables because the remaining significant variables overlap in content or have too many categories for regression models.

In a first step we included the control variable “area of profession” and in a second step the independent variable group (*clinicians* vs. *researchers*) to predict the answers. All models are significant with moderate effect sizes (Nagelkerke *R*^2^ between .2 and .4, see Table 5). After controlling for “area of profession”, *clinicians* and *researchers* differed in their answers to the mania/hypomania item (with *clinicians* more often agreeing with the differential diagnosis than *researchers*; see Table 1) but not to “repeated unsuccessful efforts to reduce or control buying/shopping” item.

**Table 5:** Results of the hierarchical binary logistic regressions with the control variable “area of profession and the predictor “group” to predict answers.

		DV: repeated unsuccessful efforts to reduce or control buying/shopping		DV: excessive buying/shopping does not occur exclusively during a period of mania/hypomania	
Step	Predictor	Model 1	Model 2	Model 1	Model 2
1	area of profession	*W*(5)^a^ = 12.05*	*W*(5)^a^ = 6.02	*W*(5)^a^ = 15.00**	*W*(5)^a^ = 5.23
2	group^b^		*W*(1)^a^ = 2.91		*W*(1)^a^ = 4.04*
Nagelkerkes R^2^		.173*	.214*	.282**	.365***
ΔR^2^			.041		.083*

Note. DV = dependent variable

*p < .05

**p < .01

***p < .001

^a^Wald coefficient with degrees of freedom

^b^group = *clinicians* vs. *researcher*.

We also performed a second regression model with “frequency of assessment/treatment in the last year” as control variable. Again, the groups differed in their responses to the mania/hypomania items. With regard to “repeated unsuccessful efforts to reduce or control buying/shopping”, the model did not reach significance as indicated by Nagelkerkes *R^2^* and low effect size. Thus, the significance of the predictor group should not be interpreted (see Table 6).

**Table 6:** Results of the hierarchical binary logistic regressions with the control variable “frequency of assessment/treatment in the last year” and the predictor “group” to predict answers.

		DV: repeated unsuccessful efforts to reduce or control buying/shopping		DV: excessive buying/shopping does not occur exclusively during a period of mania/hypomania	
Step	Predictor	Model 1	Model 2	Model 1	Model 2
1	frequency of assessment/treatment in the last year	*W*(4)^a^ = 1.05	*W*(4)^a^ = 1.64	*W*(4)^a^ = 2.11	*W*(4)^a^ = 0.85
2	group^b^		*W*(1)^a^ = 7.48**		*W*(1)^a^ = 7.83**
Nagelkerkes R^2^		.029	.153	.058	.273**
ΔR^2^			.124*		.215***

Note. DV = dependent variable; *p < .05; **p < .01; ***p < .001; ^a^Wald coefficient with degrees of freedom; ^b^group = *clinicians* vs. *researcher*.

## Discussion

A detailed discussion of the proposed diagnostic criteria for CBSD and the Delphi study has been provided when reporting the primary findings of the Delphi study [3]. The most significant result of this secondary analysis refers to the fact that responses from the two groups to importance ratings of proposed diagnostic criteria, differential diagnosis, and specifiers of CBSD converged with only few minor differences. Only the assessment of the importance of the diagnostic criterion “repeated unsuccessful efforts to reduce or control buying/shopping” and the differential diagnosis “mania/hypomania” differed between groups, with *clinicians* more often agreeing with these characteristics than *researchers*. These differences should not be overinterpreted because of the small to moderate effect sizes. Moreover, in both groups at least 75% of participants rated the criteria/differential diagnosis as important/essential (i.e., both groups agreed with them). Nonetheless, we shed more light on the result by accounting for demographic differences between the two groups, particularly their main area of profession and their experience in assessing/treating individuals with CBSD. Regarding the importance of the criterion “repeated unsuccessful efforts to reduce or control buying/shopping”, the post hoc analyses suggest that the group differences were influenced by the area of profession as well as the frequency of contact with individuals with CBSD. One reason for this could be that experts who have frequent contact with patients experiencing CBSD (e.g., in the field of medicine or clinical psychology) place a greater focus on patients’ motivation to overcome addictive behaviors than experts with less frequent contact (e.g., in the field of consumer research). With respect to the mania/hypomania item, the post-hoc analyses support the previous results regarding the group differences. It appears that *clinicians* consider this differential diagnosis very important regardless of their field of work or number of contacts with individuals with CBSD. This fits with the first attempts at defining CBSD in the early 1990 years, which emphasized the differentiation of compulsive buying from mania/hypomania [2]. *Researchers* seem to find the delineation less important.

With regards to the various hoarding items, the two groups did not differ significantly in response frequencies. While this also applies to the specifier “with vs. without hoarding disorder”, it is interesting that the consensus criterion (i.e., at least 75% of the group had to rate the specifier as important/essential) was reached by *clinicians* (78.2%) but not by *researchers* (70.6%) group. *Clinicians* may have met the consensus threshold because that group might be more sensitive to patients’ impairments caused by excessive hoarding. The accumulation of purchased goods that results in living spaces becoming cluttered and the difficulty discarding possessions reported by a subgroup of patients with CBSD interferes with their functioning in daily life and therapeutic success [27,28]. It appears that the influence of hoarding symptoms was more often considered in studies with patients seeking treatment for CBSD [28,29] than in, for example, neuropsychological studies in non-clinical, convenience samples [30]. However, the consideration of excessive hoarding in patients with CBSD would also be important in neuropsychological studies, given that cognitive performance of individuals with CBSD and hoarding symptoms may be impacted by hoarding-related cognitive deficits (e.g., with regard to problem solving, attention, organization, inhibitory control, decision making; [31]. Moreover, it is interesting that the specifiers “predominantly offline” vs. “predominantly online” did not reach consensus (less than 75% agreement) among both *clinicians* and *researchers*, without significant difference between the groups. It could be argued that patients with predominantly online behavior are not yet visible in research and clinical practice because CBSD due to the treatment seeking delay [32,33].

It is worth mentioning that *clinicians* rated their work experience related to CBSD higher than *researchers*, although the magnitude of this group effect was again only moderate (see Table 1). On the one hand, this can be explained with the high prevalence of CBSD [7] and the fact that patients with compulsive buying-shopping problems have presented for treatment over many years [2,34–36], resulting in more awareness and experience with respective patients among many clinicians. On the other hand, at least for a while, the pending recognition of CBSD as a separate mental disorder may have hindered the implementation of research projects [37], leading to less practical experience with individuals experiencing CBSD among some researchers. It should be noted that the number of research projects on CBSD, including experimental studies, has increased in recent years [12,14,38].

There are some limitations which have to be considered when interpreting the current findings. Due to the allocation of experts into *clinicians* and *researchers* based on their response to a single question regarding their primary field of work, it cannot be excluded that experts were incorrectly allocated by the authors. Perhaps some participants work both clinically and scientifically (e.g., in university hospitals/clinics). Thus, splitting the expert panel into two groups might have been somewhat artificial. However, the group differences in responses to the questions “For how many years have you been treating or assessing individuals with BSD?” and “In the past 12 months, how often have you treated or assessed individuals with BSD?” (see Table 1) and the high amount of consumer researchers in the *researchers* group compared to the *clinicians* group indicate that the latter group comprised of more experts with primarily clinical background. This supports the validity of the group allocation. Another concern refers to the fact that most experts were from either Germany or the United States. Therefore, our findings may not necessarily be suitable for all cultures or minority groups.

## Conclusions

To our knowledge, this is the first study which examined differences in ratings of diagnostic criteria for CBSD between *clinicians* and *researchers*. The reasonable sample size of the Delphi study allowed the division of the expert panel into these two groups. The high level of agreement between experts working *clinically* or within a *research* context shows that the proposed diagnostic criteria for CBSD can provide a good basis for further projects in both areas. The high level of agreement between *clinicians* and *researchers* in the Delphi study could further contribute to a high level of acceptance of the proposed criteria for CBSD and serve as a basis for the assessment of affected patients and future studies. In summary, we assume that the preliminary diagnostic criteria based on the international expert consensus offer important starting points for further research and a helpful perspective for clinical practice. The clinical applicability and diagnostic validity of the proposed criteria should now be investigated in field studies to support the recognition of CBSD as a distinct mental disorder.
